# Bacterial community composition of the sediment in Sayram Lake, an alpine lake in the arid northwest of China

**DOI:** 10.1186/s12866-023-02793-1

**Published:** 2023-02-23

**Authors:** Keqiang Shao, Lei Zhang, Tunasheng Ba, Jianying Chao, Guang Gao

**Affiliations:** 1 School of Civil Engineering and Architecture, Chuzhou University, Chuzhou, 239099 China; 2 Nanjing Institute of Geography and Limnology, Chinese Academy of Sciences, Nanjing, 210008 China; 3 Environmental Monitoring Station of Bayingolin Mongolia Autonomous Prefecture, Korle, 841000 China; 4 Nanjing Institute of Environmental Sciences, Ministry of Ecology and Environment, Nanjing, 210042 China

**Keywords:** Sayram Lake, Surface sediment, BCC, TOC, Illumina Miseq sequencing

## Abstract

Sediment bacterial communities play a critical role in biogeochemical cycling in alpine lake ecosystems. However, little is known about the sediment microbial communities in these lakes. In this study, the bacterial community composition (BCC) and their relationships with environmental factors of the sediment in Sayram Lake, the largest alpine and cold-water inland lake, China was analyzed using Illumina MiSeq sequencing. In total, we obtained 618,271 high quality sequences. The results showed that the bacterial communities with 30 phyla and 546 genera, were spread out among the 5 furface sediment samples, respectively. The communities were dominated by Proteobacteria, Acidobacteria, Planctomycetes, Gemmatimonadetes, Chloroflexi, Actinobacteria, Verrucomicrobia and Bacteroidetes, accounting for 48.15 ± 8.10%, 11.23 ± 3.10%, 8.42 ± 2.15%, 8.37 ± 2.26%, 7.40 ± 3.05%, 5.62 ± 1.25%, 4.18 ± 2.12% and 2.24 ± 1.10% of the total reads, respectively. At the genus level, the communities were dominated by *Aquabacterium*, *Pseudomonas*, *Woeseia*, *MND1*, *Ignavibacterium* and *Truepera*, accounting for 7.89% ± 8.24%, 2.32% ± 1.05%, 2.14% ± 0.94%, 2% ± 1.22%, 0.94% ± 0.14% and 0.80% ± 0.14% of the total reads, respectively. Statistical analyses showed the similarity of the sediment bacterial communities at our field sites was considerably low, far below 35%, and total organic carbon (TOC) was the dominant environmental factor affecting the spatial changes of BCC in the sediment. Thus, this study greatly improving our understanding of the microbial ecology of alpine lake in the arid and semi-arid ecosystems today so seriously threatened.

**Supplementary Information:**

The online version contains supplementary material available at 10.1186/s12866-023-02793-1.

## Introduction

Sediment bacterial communities are a major constituent of lake ecosystems, where they play a critical role in in decomposition of organic matter and mineralization of nutrients [1]. Recently, lakes, especially those lakes in arid and semi-arid regions, have been described as early indicators of both regional and global environmental change, and bacteria are thought to be a sensitive sentinel to those environmental changes [2–4]. Therefore, within lake environments, investigation of the sediment bacterial community can provide important clues to understanding ecosystem processes.

Determining the bacterial diversity and community composition in different ecotype lakes is one of the necessary steps in understanding aquatic microbial ecology. The 16S ribosomal ribonucleic acid (rRNA) gene-based high-throughput sequencing now provide us with unprecedented access to the diversity and composition of lake bacterial community, and have enabled us to identify the numerically dominant species in these ecosystems and learn much about their distributions in time and space [5–7]. Most of our knowledge about lake BCC originates from investigations of lowland freshwater lake systems [8–12]. Only recently have similar studies from saline and hypersaline habitats been reported [13–16]. Indeed, very few studies of microbial ecology have been performed in alpine lakes, especially those in arid and semi-arid regions.

Arid and semi-arid regions compose around 33% of the world’s land area, and in China the percentage is considerably higher, 52.5%. Lakes in these regions provide sparse, but valuable, water resources for human beings [17]. The Tianshan Mountains lie in Central Asia and extend over Xinjiang Uygur autonomous region, China, Kazakhstan, and Kirghizstan, with a total length of 2500 km, running east–west and a width ranging from 250 to 400 km [18]. Sayram Lake, in the arid northwest of China, is an alpine lake in the center of the Tianshan Mountains. Although there are detailed studies on water quality and paleolimnology of Sayram Lake, very little is known about the sediment bacterial communities inhabiting in the lake.

The present study is the first to use Illumina MiSeq sequencing of 16Sr RNA for the sediment microbial communities in Sayram Lake. Our aims were to: (i) characterize the BCC of the surface sediment in this alpine lake, (ii) determine which major environmental factor control the spatial changes of the sediment BCC.

## Materials and methods

### Study area

**Fig. 1:**
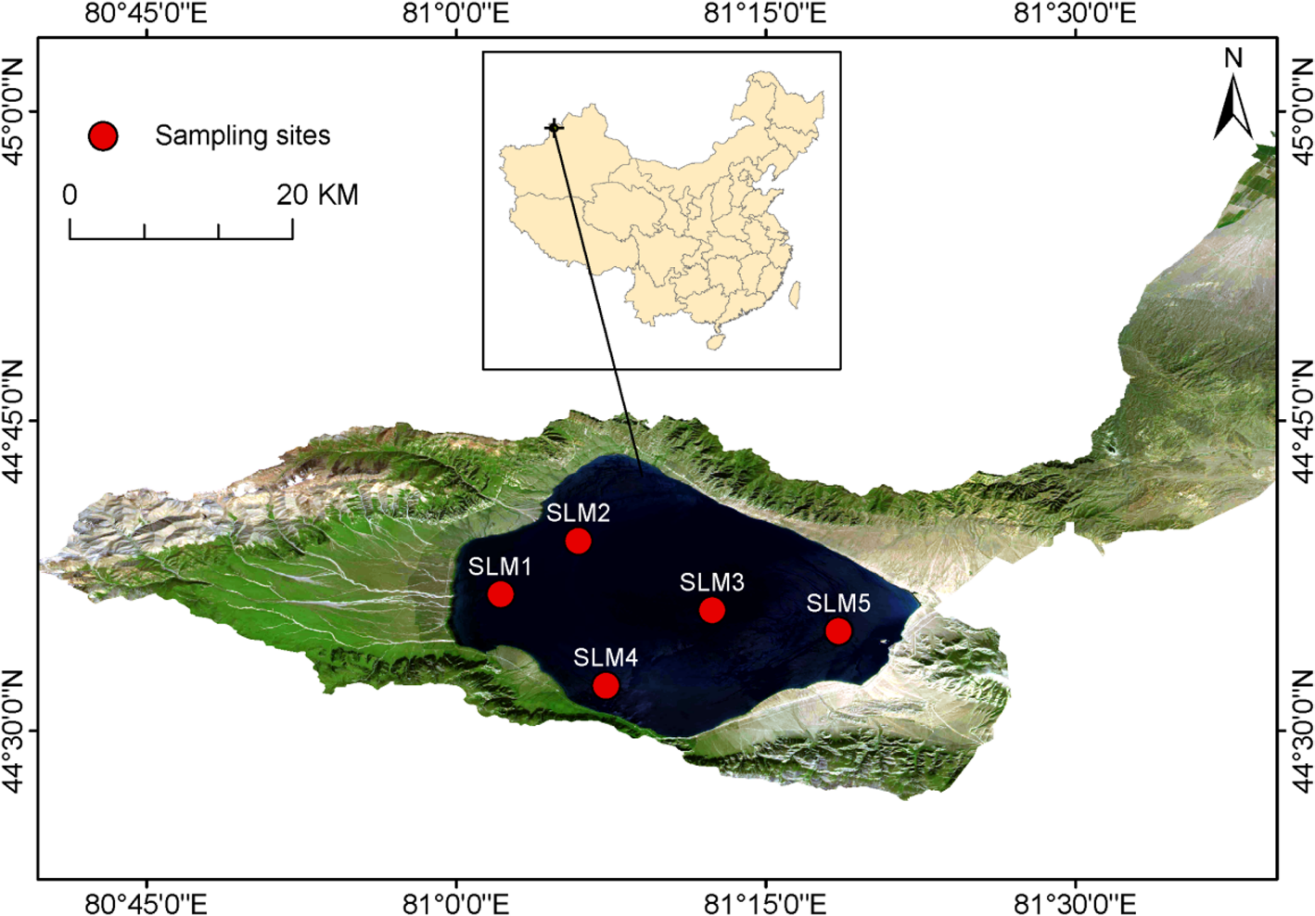
Map of Sayram Lake basin, China, showing the location of 5 sampling sites (SLM1, SLM2, SLM3, SLM4, SLM5)

Sayram Lake, is the largest alpine lake and the highest (2073 m above mean sea level) cold water inland lake in Xinjiang Uygur autonomous region, China (Fig. 1). The surface area of lake is 458 km^2^, water mean temperature is 1.1 °C and highest temperature is about 19 °C. Only a few small rivers flows into Sayram Lake and water input mainly originate from groundwater recharge [18]. Within the lake, we worked at 5 sampling sites — SLM1, SLM2, SLM3, SLM4, SLM5 (Fig. 1).

### Sampling and geochemistry measurements

Three surface sediment (0–2 cm), was collected with peterson sediment sampler, from each sampling sites on 16 August 2018. The pH was measured in situ with specific electrodes (REX, PHB-5, INESA, China). The salinity was measured on location by soil electrical conductivity meter (HI98331, HANNA, China). The sediment samples were immediately transferred into sterile plastic containers, and dried with a freeze dryer (LABCONCO, USA) in the labortaory. Total nitrogen (TN) was determined by the micro-Kjeldahl method and total phosphorus (TP) was measured by Mo-Sb colorimetric method. TOC content and C/N ratios were analyzed using an elemental analyzer (Vario EL III, Germany).

### DNA extraction and Illumina Miseq sequencing

Sediment DNA was extracted from 0.5 g sample (dry weight) using the MoBio PowerSoil™ DNA isolation kit (MoBio Laboratories Inc, Carlsbad, CA, USA) following the instructions of the manufacturer. The V3-V4 hypervariable regions of the bacterial 16S rRNA genes were polymerase chain reaction (PCR) amplified using the primer set 338F (5′-ACTCCTACGGGAGGCAGCAG-3′) and 806R (5′-GGACTACHVGGGTWTCTAAT-3′) [19]. Equimolar amounts of barcoded amplicons for each sample were sequenced using the Illumina MiSeq platform by the Shanghai Personal Biotechnology (Co. Ltd.), China.

### Sequences processing and analysis

The bioinformatic analysis was performed using the Microbial Genomics Module on CLC Genomics Workbench 20.0 (Qiagen, Aarhus C, Denmark). After importing raw reads, a standard quality control process was implemented, comprising merger paired reads (minimum overlap of 200 bp), trim off adapters and the primer sequences. Microbial phylotypes were assigned to operational taxonomic units (OTUs, 97% similarity). The low abundance OTUs (< 10 reads) was filtered out for minimizing the random sequencing error. Taxonomic classification of the sequences was conducted using the SILVA small subunit rRNA (SSU) database v132 [20]. Sequences related with chimeras and chloroplast was excluded from subsequent analysis. The α-diversity, including Shannon, Simpson, Chao1 and ACE indexes was analysed using the Mothur software (version 1.31.2).

### Statistical analyses

The statistical analyses and the visualization were performed by Microsoft Excel. To evaluate the β-diversity (variation of community structures) among the 5 surface sediment samples, we performed cluster analysis and non-metric multidimensional scaling (NMDS) by using Bray–Curtis similarity based on distance matrix of bacterial community [21, 22]. To analyze the significant environmental variable associated with BCC, we carried out Canonical correspondence analysis (CCA) using CANOCO 5.0 (Microcomputer Power, Ithaca NY, USA).

## Results

### Physicochemical characteristics

The longitude and latitude of sampling sites, and the value for physicochemical parameters of the surface sediment in Sayram Lake is summarized in Table S1. The pH values for sediment ranged from 9.11 (SLM2) to 9.13 (SLM5), and salinity ranged from 2.538 ms/cm (SLM1) to 2.548 ms/cm ((SLM4). The contents of TN and TP for sediment ranged from 0.513% to 0.546%, 0.069% to 0.071%, respectively, with the lowest value found in site SLM2, and the highest in site SLM5. Sediment TOC content, and C/N ratio varied from 4.854 (SLM1) to 5.014% (SLM4), and 8.859 (SLM1) to 9.045 (SLM2), respectively*.*

### Bacterial α-diversity and β-diversity

We generated 618,271 high-quality reads with a mean of 123,654 reads per sample. Across the 5 different surface sediment samples, the reads were classified into 2,486 OTUs. The rarefaction curves of Shannon index approached an asymptote after 1,500 reads (Fig. S1), indicating enough sequencing depth for further analysis. This was confirmed by the high Good’s coverage, ranging from 98.90%to 99.53% (Table S2).

The bacterial α-diversity patterns, including the Shannon, Simpson, Chao1 and ACE indexes were distinct among the 5 surface sediments, with the lowest value found in site SLM4, and the highest in site SLM1 (Table S2). For the Shannon and Simpson indices, α-diversity (community diversity) ranged from 6.4535 to 7.5702, and 0.9516 to 0.9874, respectively. For the Chao-1 and ACE indices, α-diversity (community richness) ranged from 732 to 904, and 495 to 634, respectively.

**Fig. 2:**
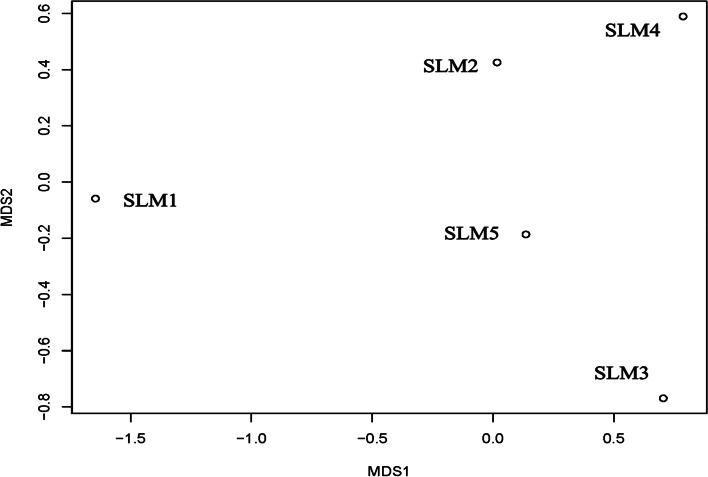
The NMDS plot based on the UniFrac weighted distance calculated from read numbers among the 5 sampling sites in Sayram Lake

We investigated the spatial β-diversity patterns of the sediment bacterial communities by using NMDS. Notably, the sediment samples were scattered on the NMDS plot, suggesting low similarity of the sediment BCC among the 5 different sampling sites (Fig. 2).

### Bacterial taxonomy and community structure

**Fig. 3:**
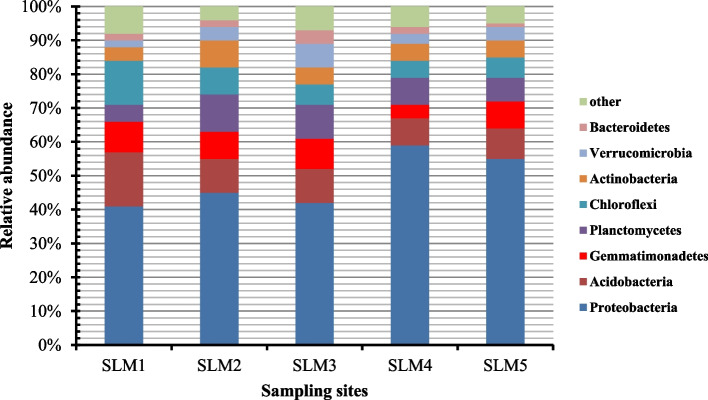
The relative abundance of BCC at the phylum level in the surface sediment of Sayram Lake

**Fig. 4:**
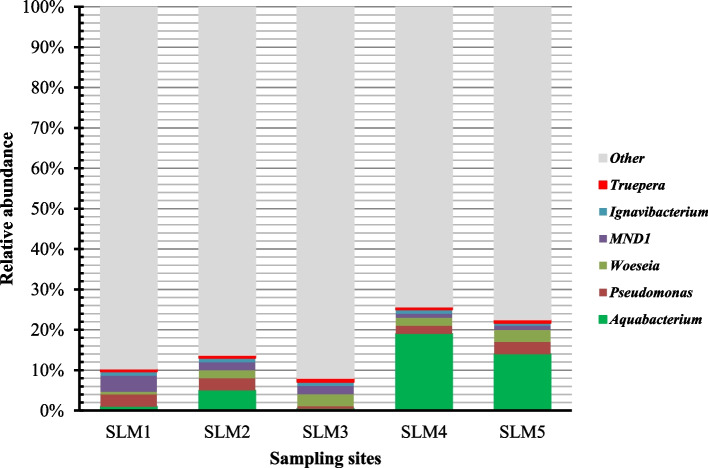
The relative abundance of BCC at the genus level in the surface sediment of Sayram Lake

In the Sayram lake sediment habitats, we detected a total of 30 different phyla: the dominant phyla (mean relative abundance ≥ 2%) were Proteobacteria, Acidobacteria, Planctomycetes, Gemmatimonadetes, Chloroflexi, Actinobacteria, Verrucomicrobia and Bacteroidetes, accounting for 48.15 ± 8.10%, 11.23 ± 3.10%, 8.42 ± 2.15%, 8.37 ± 2.26%, 7.40 ± 3.05%, 5.62 ± 1.25%, 4.18 ± 2.12% and 2.24 ± 1.10% of the total reads, respectively (Fig. 3). At the genus level, we detected a total of 546 different genus: the 6 dominant genus (mean relative abundance ≥ 0.8%) were *Aquabacterium*, *Pseudomonas*, *Woeseia*, *MND1*, *Ignavibacterium* and *Truepera*, accounting for 7.89% ± 8.24%, 2.32% ± 1.05%, 2.14% ± 0.94%, 2% ± 1.22%, 0.94% ± 0.14% and 0.80% ± 0.14% of the total reads, respectively (Fig. 4). To determine in more detail the similarities of the bacterial communities in surface sediment, cluster analysis was carried out. The resulting dendrograms indicated the BCC among 5 different sediment samples is of low similarity, only with the similarities 20–35% (Fig. S2).

### Relationships of environmental factors and sediment bacterial communities

**Fig. 5:**
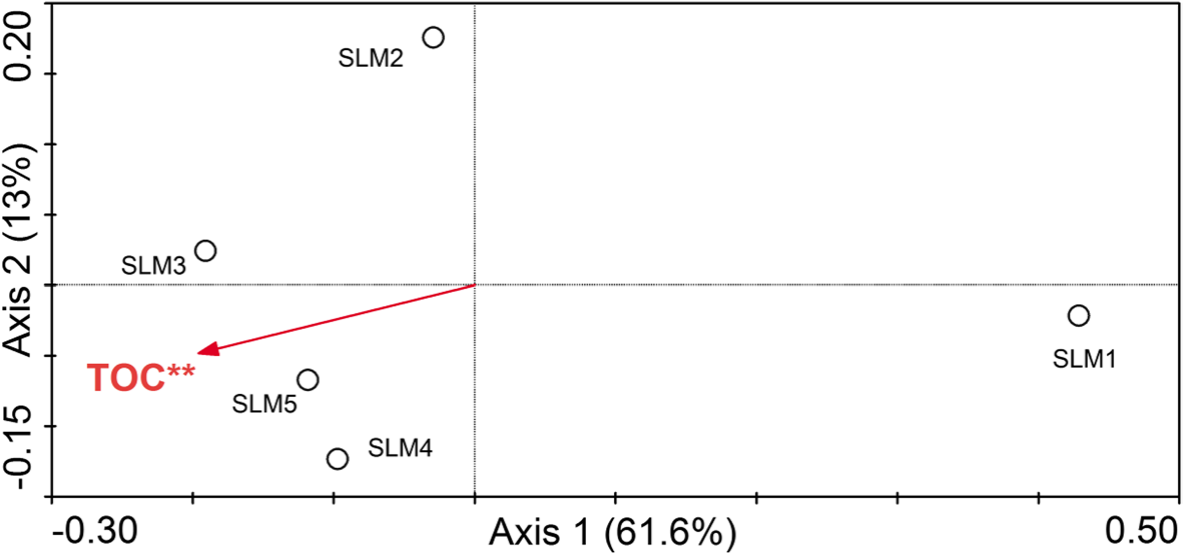
The CCA biplot showed the relationship between environmental factors and sediment bacterial community. The statistically significant variable is marked with an asterisk (**) according to a Monte Carlo permutation test (*P* < 0.01). Abbreviations:TOC, Total organic carbon

We prepared a CCA biplot of the sediment BCC and the 6 environmental variables (TN, TP, pH, C/N, TOC and Salinity) for Sayram Lake (Fig. 5). The plot showed that TOC was the significant environmental variable affecting the spatial changes of BCC in the sediment. The eigenvalues of the first and second axis were 0.049 and 0.01, respectively, and these 2 axes explained 74.6% of the spatial variation in the BCC of the sediment.

## Discussion

### Sediment BCC in Sayram Lake

In this study, we have shown that the dominant bacterial phylum in the Sayram Lake sediment habitats were Proteobacteria, Acidobacteria, Planctomycetes, Gemmatimonadetes, Chloroflexi, Actinobacteria, Verrucomicrobia, Bacteroidetes, and these phyla are found in the waters and sediments of other various lakes. It is known that Proteobacteria is the largest phylum of bacteria, and they might be involved in the functioning and processes of lake ecosystems [23, 24]. Proteobacteria was commonly observed in waters and sediments from other saline and alkaline lakes [25–28] and marine coastal waters [29]. Acidobacteria have the ability of their resistance to antibiotics, and can utilize a wide variety of simple and complex carbohydrates as substrates, and play important roles in the C, N and S biogeochemical cycles [30]. They were not only found in the saline and alkaline Qinghai Lake, China [31], but also dominanted in 13 freshwater lakes sediment on the Yunnan Plateau, China [32]. Planctomycetes have the ability to degrade complex sulfated polysaccharides of algal origin [33, 34]. They are mainly characterized by an attached lifestyle, adhering to surfaces in the various environments include aquatic, extreme and polluted habitats [34]. Gemmatimonadetes is a new phylum, is defined on a phylogenetic basis by comparative 16S rDNA sequence analysis of one isolated strain and uncultured representatives from multiple terrestrial and aquatic habitats [24]. They were found as the major components of sediment bacterial communities in 13 freshwater lakes on the Yunnan Plateau, China [32]. Chloroflexi was ubiquitous in the environment, and they were found in sediments of lakes [32, 35–37], and the hot springs in the Tibetan Plateau, China [38]. Actinobacteria was commonly a minor bacterial group or was even absent in lake sediment ecosystems [23, 35, 39, 40]. But Actinobacteria was also identified as the major components of sediment bacterial communities in 13 freshwater lakes on the Yunnan Plateau, China [32]. Verrucomicrobia are capable of degrading diverse polysaccharides [41] and fixing nitrogen [42, 43]. They were also the major components of sediment bacterial communities in 13 freshwater lakes on the Yunnan Plateau, China [32]. Bacteroidetes was reported to be associated with nutrient conversion in lake sediments [44]. So far, previous studies reported the dominance of Bacteroidetes in freshwater lake sediment [36, 39], inland lakes [14, 16, 44] and estuaries [43, 45–47].

The dominant genus of Sayram Lake sediment were *Aquabacterium*, *Pseudomonas*, *Woeseia*, *MND1*, *Ignavibacterium*, and *Truepera*, and these genera also existed in other various environments. Previous study have showed that *Aquabacterium* dominated in the high arsenic groundwater, and *Pseudomonas* dominated in the high arsenic sediments of Hetao Plain of Inner Mongolia, China [48]. *Pseudomonas* was also found in sediments of saline Qinghai Lake [31], and Lake Chaka, a hypersaline lake on Tibetan plateau, China [49]. *Woeseia* has previously been reported to existed in offshore sediments from Shenzhen, China [50]. *Woeseia* and *Ignavibacterium* were also greatly enriched in saline–sodic soils, Northeast China [51]. Ya et al. [52] have indicated that *Ignavibacterium* was the putative keystone species, could be responsible for the deterioration of nitrogen removal under high salinity condition in wastewater treatment system. Furthermore, Shen et al. [53] have reported that the *MND1* was existed in petroleum-contaminated soils. In addition, *Truepera* has been found in environments as diverse as stone ruins at historic sites in Tamil Nadu, India [54], an iron tailing pond in northwest China [55] and composting distilled grain waste [56].

### Environmental factors governing sediment bacterial communities

Our CCA results suggested that TOC significantly influenced the bacterial community structure of the sediment in Sayram Lake, which is in agreement with previous studies of various environments. In Lake Chaohu, China, TOC has a significant effect on the BCC of two estuaries of this lake [57]. In Lake Bosten, China, TOC was the main environmental factor influencing the sediment bacterial community [38]. TOC was also the most important driving factor that determined the bacterial community in urban river sediments [57]. Furthermore, TOC concentration statistically explained the differences in the bacterial diversity and community composition within different sampling sites in the coastal aquaculture area [58]. Zhang et al. [59] have indicated that TOC plays an important role in shaping the microbial community structure in these groundwater systems in Qiji County, Yuncheng City, China. In addition, TOC was also the major factor in the open areas driving bacterial communities in water of the Xixi National Wetland Park, China [60]. Numerous studies have shown that carbon, either organic carbon or inorganic carbon, is a necessary nutrient element for microorganisms and is one of the fundamental factors that structures microbial communities by controlling the growth and distribution of microorganism [59].

## Supplementary Information

**Additional file 1: Table S1.** The longitude and latitude of sampling sites, and the value for physicochemical parameters of 5 surface sediments in Sayram Lake. **Table S2.** The OTU-based species diversity represented by 4 α-diversity indices and Goods coverage of 5 sediment samples in Sayram Lake. **Fig. S1.** Rarefaction curves for the different sediment samples analyzed using the Shannon diversity. **Fig. S2.** Sediment bacterial communities clustering generated from the 16S ribosomal ribonucleic acid (rRNA) gene-based Illumina MiSeq sequencing of 16S rDNA OTUs data (97% similarity).

## Data Availability

The raw sequencing data in our study have been deposited at the Sequence Read Archive database of the National Center for Biotechnology Information (NCBI) with the BioProject accession number PRJNA888417 (GenBank BioSample identifier; SLM1, SAMN31217440; SLM2, SAMN31217441; SLM3, SAMN31217442; SLM4, SAMN31217443; SLM5, SAMN31217444).

## References

[1] Roberto AA, Van Gray JB, Leff LG (2018). Sediment bacteria in an urban stream: spatiotemporal patterns in community composition. Water Res.

[2] Williamson CE, Dodds W, Kratz TK, Palmer MA (2008). Lakes and streams as sentinels of environmental change in terrestrial and atmospheric processes. Front Ecol Environ.

[3] Newton RJ, Jones SE, Eiler A, McMahon KD, Bertilsson S (2011). A guide to the natural history of freshwater lake bacteria. Microbiol Mol Biol Rev.

[4] Adrian R, O’Reilly CM, Zagarese H, Baines SB, Hessen DO, Keller W, Livingstone DM, Sommaruga R, Straile D, Donk EV, Weyhenmeyer GA, Winder M (2009). Lakes as sentinels of climate change. Limnol Oceanogr.

[5] Curtis TP, Sloan WT (2004). Prokaryotic diversity and its limits:microbial community structure in nature and implications for microbial ecology. Curr Opin Microbiol.

[6] Kent AD, Yannarell AC, Rusak JA, Triplett EW, McMahon KD (2007). Synchrony in aquatic microbial community dynamics. ISME J.

[7] Shade A, Jones SE, McMahon KD (2008). The influence of habitat heterogeneity on freshwater bacterial community composition and dynamics. Environ Microbiol.

[8] Zwart G, Crump BC, Agterveld MPK, Hagen F, Han SK (2002). Typical freshwater bacteria:an analysis of available 16S rRNA gene sequences from plankton of lakes and rivers. Aquat Microb Ecol.

[9] Eiler A, Bertilsson S (2004). Composition of freshwater bacterial communities associated with cyanobacterial blooms in four Swedish lakes. Environ Microbiol.

[10] Van der Gucht K, Vandekerckhove T, Vloemans N, Cousin S, Muylaert K, Sabbe K, Gillis M, Declerk S, Meester LD, Vyverman W (2005). Characterization of bacterial communities in four freshwater lakes differing in nutrient load and food web structure. FEMS Microbiol Ecol.

[11] Wu QL, Zwart G, Wu J, Agterveld M, Liu S, Hahn MW (2007). Submersed macrophytes play a key role in structuring bacterioplankton community composition in the large, shallow, subtropical Taihu Lake, China. Environ Microbiol.

[12] Tang XM, Gao G, Chao JY, Wang XD, Zhu GW, Qin BQ (2010). Dynamics of organic-aggregate-associated bacterial communities and related environmental factors in Lake Taihu, a large eutrophic shallow lake in China. Limnol Oceanogr.

[13] Jiang HC, Dong HL, Zhang GX, Yu BS, Chapman LR, Fields MW (2006). Microbial diversity in water and sediment of Lake Chaka, an athalassohaline lake in northwestern China. Appl Environ Microbiol.

[14] Wu QL, Zwart G, Schauer M, Kamst-van Agterveld MP, Hahn MW (2006). Bacterioplankton community composition along a salinity gradient of sixteen high-mountain lakes located on the Tibetan Plateau. China Appl Environ Microbiol.

[15] Tian C, Tan J, Wu X, Ye WJ, Liu XL, Li DT, Yang H (2009). Spatiotemporal transition of bacterioplankton diversity in a large shallow hypertrophic freshwater lake, as determined by denaturing gradient gel electrophoresis. J Plankton Res.

[16] Xing P, Hahn MW, Wu QL (2009). Low taxon richness of bacterioplankton in high-altitude lakes of the eastern Tibetan Plateau, with a predominance of Bacteroidetes and Synechococcus spp. Appl Environ Microbiol.

[17] Bai J, Chen X, Li J, Yang L, Fang H (2011). Changes in the area of inland lakes in arid regions of central Asia during the past 30 years. Environ Monit Assess.

[18] Zeng J, Deng LJ, Lou K, Zhang T, Yang HM, Shi YW, Lin Q (2014). Molecular characterization of the planktonic microorganisms in water of two mountain brackish lakes. J Basic Microbiol.

[19] Fadrosh DW, Ma B, Gajer P, Sengamalay N, Ott S, Brotman RM, Ravel J (2014). An improved dual-indexing approach for multiplexed 16S rRNA gene sequencing on the Illumina MiSeq platform. Microbiome.

[20] Quast C, Pruesse E, Yilmaz P, Gerken J, Schweer T, Yarza P, Peplies J, Glöckner FO (2013). The SILVA ribosomal RNA gene database project: Improved data processing and web-based tools. Nucleic Acids Res.

[21] Clarke KR (1993). Non-parametric multivariate analyses of changes in community structure. Aust J Ecol.

[22] Borcard DH, Tyson GW, Hugenholtz P, Beiko RG (2014). STAMP: Statistical analysis of taxonomic and functional profiles. Bioinformatics.

[23] Ye WJ, Liu XL, Lin SQ, Tan J, Pan JL, Li DT, Yang H (2009). The vertical distribution of bacterial and archaeal communities in the water and sediment of Lake Taihu. FEMS Microbiol Ecol.

[24] Zhang JX, Zhang XL, Liu Y, Xie SG, Liu YG (2013). Bacterioplankton communities in a high-altitude freshwater wetland. Ann Microbiol.

[25] Humayoun SB, Bano N, Hollibaugh JT (2003). Depth distribution of microbial diversity in Mono Lake, a meromictic soda lake in California. Appl Environ Microbiol.

[26] Demergasso C, Casamayor EO, Chong G, Galleguillos P, Escudero L, Pedros-Alio C (2004). Distribution of prokaryotic genetic diversity in athalassohaline lakes of the Atacama Desert, Northern Chile. FEMS Microbiol Ecol.

[27] Koizumi Y, Kojima H, Fukui M (2004). Dominant microbial composition and its vertical distribution in saline meromictic Lake Kaiike (Japan) as revealed by quantitative oligonucleotide probe membrane hybridization. Appl Environ Microbiol.

[28] Rees HC, Grant WD, Jones BE, Heaphy S (2004). Diversity of Kenyan soda lake alkaliphiles assessed by molecular methods. Extremephiles.

[29] Fortunato CS, Herfort L, Zuber P, Baptista AM, Crump BC (2012). Spatial variability overwhelms seasonal patterns in bacterioplankton communities across a river to ocean gradient. ISME J.

[30] Kuramae EE, de Assis Costa OY (2019). Acidobacteria in Encyclopedia of Microbiology (Fourth Edition).

[31] Dong HL, Zhang GX, Jiang HC, Yu BS, Chapman LR, Lucas CR, Fields MW (2006). Microbial diversity in sediments of saline Qinghai lake, China:linking geochemical controls to microbial ecology. Microb Ecol.

[32] Zhang JX, Yang YY, Zhao L, Li YZ, Xie SG, Liu Y (2015). Distribution of sediment bacterial and archaeal communities in plateau freshwater lakes. Appl Microbiol Biotechnol.

[33] Kim JY, Yoon JH, Bae JW (2008). Characterization of the depthrelated changes in the microbial communities in Lake Hovsgol sediment by 16S rRNA gene-based approaches. J Microbiol.

[34] Lage OM, Niftrik LV, Jogler C, Devos DP (2019). Planctomycetes in Encyclopedia of Microbiology (Fourth Edition).

[35] Swan BK, Ehrhardt CJ, Reifel KM, Moreno LI, Valentine DL (2010). Archaeal and bacterial communities respond differently to environmental gradients in anoxic sediments of a California hypersaline lake the Salton Sea. Appl Environ Microbiol.

[36] Bai YH, Shi Q, Wen DH, Li ZX, Jefferson WA, Feng CP, Tang XY (2012). Bacterial communities in the sediments of Dianchi Lake a partitioned eutrophic waterbody in China. PLoS ONE.

[37] Lucheta AR, Otero XL, Macias F, Lambais MR (2013). Bacterial and archaeal communities in the acid pit lake sediments of a chalcopyrite mine. Extremophiles.

[38] Zhang YM, Wu G, Jiang HC, Yang J, She WY, Khan I, Li WJ (2018). Abundant and rare microbial biospheres respond differently to environmental and spatial factors in Tibetan hot springs. Front Microbiol.

[39] Haller L, Tonolla M, Zopfi J, Peduzzi R, Wildi W, Pote J (2011). Composition of bacterial and archaeal communities in freshwater sediments with different contamination levels (Lake Geneva Switzerland). Water Res.

[40] Shivaji S, Kumari K, Kishore KH, Pindi PK, Rao PS, Srinivas TNR (2011). Vertical distribution of bacteria in a lake sediment from Antarctica by culture-independent and culturedependent approaches. Res Microbiol.

[41] Martinez-Garcia M, Brazel DM, Swan BK, Arnosti C, Chain PSG, Reitenga KG, Xie G, Poulton NJ, Gomez ML, Masland DED (2012). Capturing single cell genomes of active polysaccharide degraders:an unexpected contribution of Verrucomicrobia. PLoS ONE.

[42] Wertz JT, Kim E, Breznak JA, Schmidt TM, Rodrigues JLM (2012). Genomic and physiological characterization of the Verrucomicrobia isolate Diplosphaera colitermitum gen. nov, sp. nov, reveals microaerophily and nitrogen fixation genes. Appl Environ Microbiol.

[43] Herlemann DP, Labrenz M, Jurgens K, Bertilsson S, Waniek JJ, Andersson AF (2011). Transitions in bacterial communities along the 2000 km salinity gradient of the Baltic Sea. ISME J.

[44] Liu FH, Lin GH, Gao G, Qin BQ, Zhang JS, Zhao GP, Zhou ZH, Shen JH (2009). Bacterial and archaeal assemblages in sediments of a large shallow freshwater lake Lake Taihu as revealed by denaturing gradient gel electrophoresis. J Appl Microbiol.

[45] Crump BC, Hopkinson CS, Sogin ML, Hobbie JE (2004). Microbial biogeography along an estuarine salinity gradient:combined influences of bacterial growth and residence time. Appl Environ Microbiol.

[46] Fortunato CS, Eiler A, Herfort L, Needoba JA, Peterson TD, Crump BC (2013). Determining indicator taxa across spatial and seasonal gradients in the Columbia River coastal margin. ISME J.

[47] Kirchman DL, Dittel AI, Malmstrom RR, Cottrell MT (2005). Biogeography of major bacterial groups in the Delaware estuary. Limnol Oceanogr.

[48] Wang YH, Li P, Li B, Webster G, Weightman JA, Jiang Z, Jiang DW, Deng YM, Wang YX (2014). Bacterial diversity and community structure in high arsenic aquifers in Hetao Plain of Inner Mongolia. China Geomicrobiol J.

[49] Jiang HC, Dong HL, Yu BS, Liu XQ, Li YL, Ji SS, Zhang CL (2007). Microbial response to salinity change in Lake Chaka, a hypersaline lake on Tibetan plateau. Environ Microbiol.

[50] Zhang R, Sun MR, Zhang HL, Zhao ZH (2021). Spatial separation of microbial communities reflects gradients of salinity and temperature in offshore sediments from Shenzhen, south China. Ocean Coast Manage.

[51] Liang XL, Wang XY, Zhang N, Li BX (2022). Biogeographical patterns and assembly of bacterial communities in saline soils of Northeast China. Microorganisms.

[52] Ya T, Du S, Li ZY, Liu SD, Zhu MH, Liu XJ, Jing ZB, Hai RT, Wang XH (2021). Successional dynamics of molecular ecological network of anammox microbial communities under elevated salinity. Water Res.

[53] Shen YY, Ji Y, Li CR, Luo PP, Wang WK, Zhang Y (2018). Effects of phytoremediation treatment on bacterial community structure and diversity in different petroleum-contaminated soils. Int J Env Res Pub He.

[54] Ennis NJ, Dharumaduri D, Bryce JG, Tisa LS (2020). Metagenome across a geochemical gradient of Indian stone ruins found at historic sites in Tamil Nadu. India Microb Ecol.

[55] Li S, Wu JL, Huo YL, Zhao X, Xue LG (2020). Profiling multiple heavy metal contamination and bacterial communities surrounding an iron tailing pond in Northwest China. Sci Total Environ.

[56] Wang SP, Sun ZY, Wang ST, Yuan HW, An MZ, Xia ZY, Tang YQ, Shen CH, Kida KJ (2022). Bacterial community structure and metabolic function succession during the composting of distilled grain waste. Appl Biochem Biotechnol.

[57] Zhang L, Tu DM, Li XC, Lu WX, Li J (2020). Impact of long-term industrial contamination on the bacterial communities in urban river sediments. BMC Microbiol.

[58] Wang CX, Wang YB, Liu PY, Sun YY, Song ZL, Hu XK (2021). Characteristics of bacterial community structure and function associated with nutrients and heavy metals in coastal aquaculture area. Environ Pollut.

[59] Zhang X, Gao XB, Li CC, Luo XS, Wang YX (2019). Fluoride contributes to the shaping of microbial community in high fluoride groundwater in Qiji County, Yuncheng City. China Sci Rep-UK.

[60] Wang BH, Zheng XF, Zhang HJ, Xiao FS, Gu H, Zhang KK, He ZL, Liu X, Yan QY (2020). Bacterial community responses to tourism development in the Xixi National Wetland Park. China. Sci Total Environ.

