# Supplementary material for: Bacterial community composition of the sediment in Sayram Lake, an alpine lake in the arid northwest of China
Source: BMC Microbiol. 2023 Feb 23;23:47. doi: 10.1186/s12866-023-02793-1 (PMC9948317; doi:10.1186/s12866-023-02793-1)

**Table S1.** The longitude and latitude of sampling sites, and the value for physicochemical parameters of 5 surface sediments in Sayram Lake.

| **Samples** | **Longitude** | **Latitude** | **TN (%)** | **TOC (%)** | **C/N** | **TP (%)** | **Salinity (ms/cm)** | **pH** |
| --- | --- | --- | --- | --- | --- | --- | --- | --- |
| SLM1 | 81.036 | 44.610 | 0.517 | 4.854 | 8.859 | 0.070 | 2.538 | 9.12 |
| SLM2 | 81.099 | 44.653 | 0.513 | 4.910 | 9.038 | 0.069 | 2.540 | 9.11 |
| SLM3 | 81.207 | 44.597 | 0.528 | 4.995 | 9.044 | 0.070 | 2.541 | 9.12 |
| SLM4 | 81.121 | 44.536 | 0.542 | 5.014 | 8.981 | 0.070 | 2.548 | 9.12 |
| SLM5 | 81.309 | 44.580 | 0.546 | 5.003 | 9.001 | 0.071 | 2.542 | 9.13 |

**Table S2.** The OTU-based species diversity represented by 4 α-diversity indices and Goods coverage of 5 sediment samples in Sayram Lake.

| **Samples** | **α-diversity indices** | | | | **Goods coverage** |
| --- | --- | --- | --- | --- | --- |
|  | **Shannon** | **Simpson** | **Chao1** | **ACE** |  |
| SLM1 | 7.5702 | 0.9874 | 904 | 634 | 0.9953 |
| SLM2 | 7.0542 | 0.9801 | 751 | 521 | 0.9890 |
| SLM3 | 7.5165 | 0.9870 | 848 | 601 | 0.9916 |
| SLM4 | 6.4535 | 0.9516 | 732 | 495 | 0.9915 |
| SLM5 | 7.0849 | 0.9804 | 801 | 539 | 0.9907 |

**Figure legends**

**Fig. S1.** Rarefaction curves for the different sediment samples analyzed using the Shannon diversity.

**Fig. S2.** Sediment bacterial communities clustering generated from the 16S ribosomal ribonucleic acid (rRNA) gene-based Illumina MiSeq sequencing of 16S rDNA OTUs data (97% similarity).

**Fig. S1.**


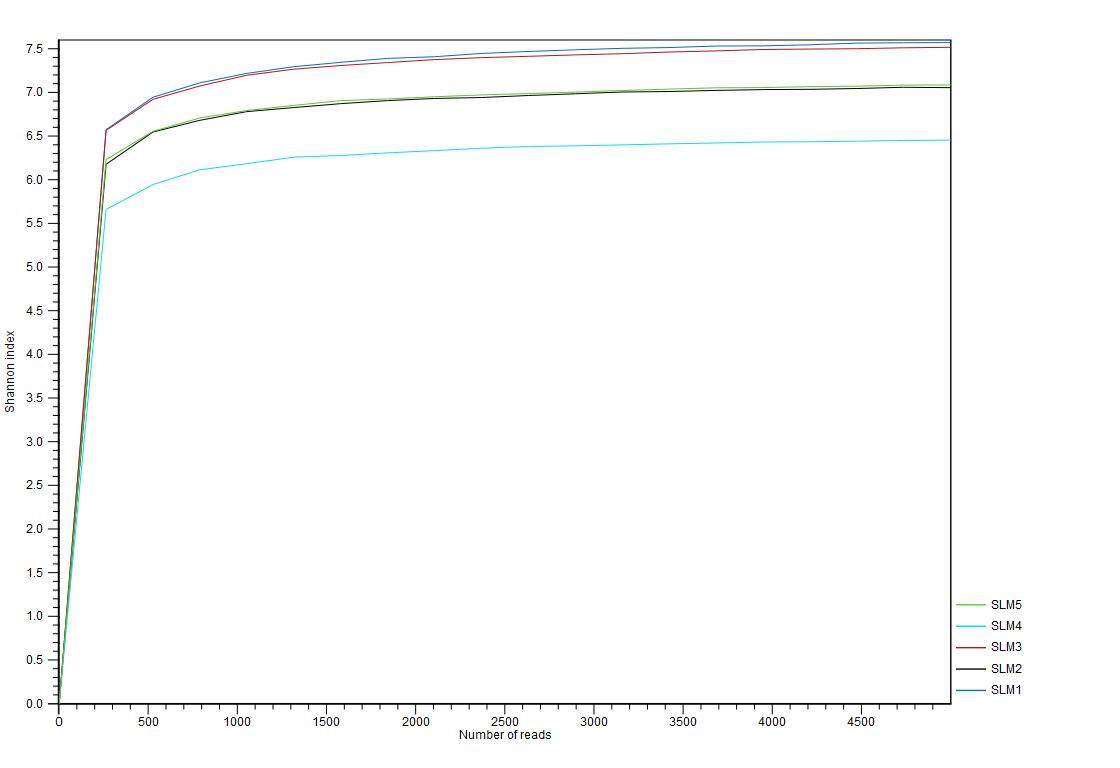


**Fig. S2.**


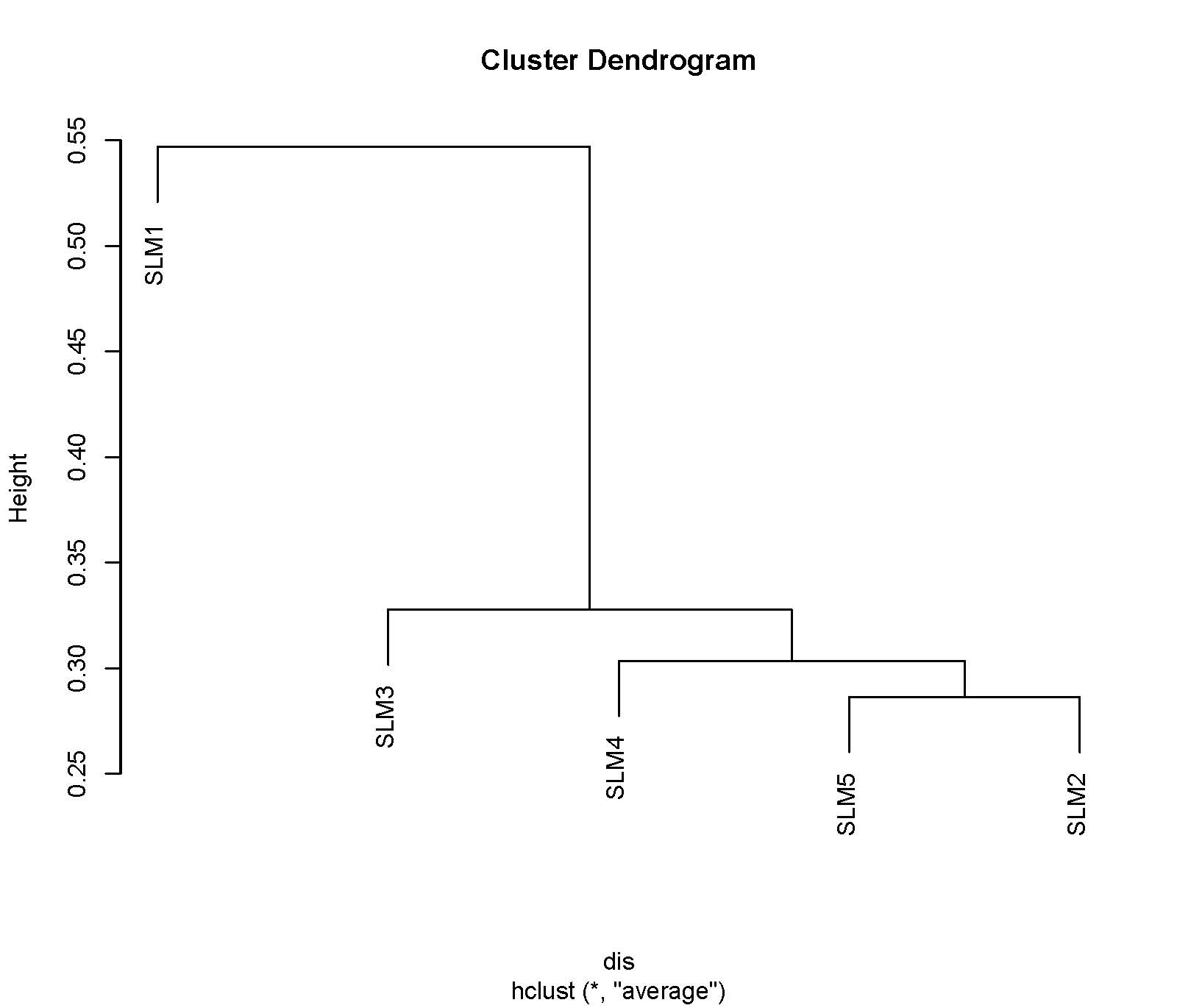

Supplement: Supplementary file 1 — Additional file 1: Table S1. The longitude and latitude of sampling sites, and the value for physicochemical parameters of 5 surface sediments in Sayram Lake. Table S2. The OTU-based species diversity represented by 4 α-diversity indices and Goods coverage of 5 sediment samples in Sayram Lake. Fig. S1. Rarefaction curves for the different sediment samples analyzed using the Shannon diversity. Fig. S2. Sediment bacterial communities clustering generated from the 16S ribosomal ribonucleic acid (rRNA) gene-based Illumina MiSeq sequencing of 16S rDNA OTUs data (97% similarity). [file 12866_2023_2793_MOESM1_ESM.docx]
